# The associations among the dopamine D2 receptor Taq1, emotional intelligence, creative potential measured by divergent thinking, and motivational state and these associations' sex differences

**DOI:** 10.3389/fpsyg.2015.00912

**Published:** 2015-07-07

**Authors:** Hikaru Takeuchi, Hiroaki Tomita, Yasuyuki Taki, Yoshie Kikuchi, Chiaki Ono, Zhiqian Yu, Atsushi Sekiguchi, Rui Nouchi, Yuka Kotozaki, Seishu Nakagawa, Carlos M. Miyauchi, Kunio Iizuka, Ryoichi Yokoyama, Takamitsu Shinada, Yuki Yamamoto, Sugiko Hanawa, Tsuyoshi Araki, Hiroshi Hashizume, Keiko Kunitoki, Yuko Sassa, Ryuta Kawashima

**Affiliations:** ^1^Division of Developmental Cognitive Neuroscience, Institute of Development, Aging and Cancer, Tohoku UniversitySendai, Japan; ^2^Department of Disaster Psychiatry, International Research Institute of Disaster Science, Tohoku UniversitySendai, Japan; ^3^Division of Medical Neuroimage Analysis, Department of Community Medical Supports, Tohoku Medical Megabank Organization, Tohoku UniversitySendai, Japan; ^4^Department of Nuclear Medicine and Radiology, Institute of Development, Aging and Cancer, Tohoku UniversitySendai, Japan; ^5^Department of Functional Brain Imaging, Institute of Development, Aging and Cancer, Tohoku UniversitySendai, Japan; ^6^Human and Social Response Research Division, International Research Institute of Disaster Science, Tohoku UniversitySendai, Japan; ^7^Smart Ageing International Research Center, Institute of Development, Aging and Cancer, Tohoku UniversitySendai, Japan; ^8^Department of General Systems Studies, Graduate School of Arts and Sciences, The University of TokyoTokyo, Japan; ^9^Department of Psychiatry, Tohoku University Graduate School of MedicineSendai, Japan; ^10^Japan Society for the Promotion of ScienceTokyo, Japan; ^11^Faculty of Medicine, Tohoku UniversitySendai, Japan

**Keywords:** emotional intelligence, mood, dopamine, creativity, divergent thinking, motivation, creative potential

## Abstract

Previous neuroscientific studies have shown that the dopaminergic system plays an important role in creative potential measured by divergent thinking (CPMDT), emotional control, and motivational state. However, although associations between two of these four components have been previously established (e.g., the association between CPMDT and emotional control, the association between CPMDT and motivational state, etc.), the interactions between these four remain unknown. The purpose of this study was to reveal these interactions using path analyses. The Taq1A polymorphism of the dopamine D2 receptor (DRD2) gene was used for this purpose. For measuring emotional intelligence (EI), we used the Japanese version of the Emotional Intelligence Scale. CPMDT was measured using the S-A creativity test. Motivational state was measured using the Vigor subscale of the Japanese version of the Profile of Mood Scale (POMS). Data from 766 healthy, right-handed individuals (426 men and 340 women; 20.7 ± 1.9 years of age) were used in this study. There were significant and robust positive relationships among measures of CPMDT, EI, and motivational state across sex. In addition, the polymorphism of the DRD2 gene was significantly associated with EI, specifically in females. Path analysis in females indicates that the model in which (a) the DRD2 polymorphism primarily facilitates EI, (b) EI in turn facilitates CPMDT and leads to a better motivational state, and (c) a better motivational state also directly facilitates CPMDT explains the data in the most accurate manner. This study suggested a comprehensive picture of the cascade of the associations among dopamine, EI, motivational state, and CPMDT at least in females.

## Introduction

The broadly accepted standard definition of creativity is the ability to produce work that is both novel and useful within a certain social context (Stein, 1953; Runco and Jaeger, 2012). Creative production has been the key to the development of our culture and civilization (Takeuchi et al., 2013a). In the laboratory setting, divergent thinking measures are widely used to measure individual differences in abilities of creative cognition (Takeuchi et al., 2011a), and they have been shown to be reliable and valid indicators of a person's creative potential (Runco and Acar, 2012; Benedek et al., 2014). Divergent thinking is defined as the generation and application of several different ideas to solve a given problem (Runco, 1990). A meta-analysis has shown that divergent thinking can strongly predict individual creativity achievement (Kim, 2008).

Psychological studies have shown that creative potential measured by divergent thinking (CPMDT) is associated with individual differences in perspectives of emotion, mood, and motivation. Creativity has been traditionally and essentially linked to motivation; specifically, it is predicted that brain motivational systems are critically relevant to creativity (for review, see Flaherty, 2005). In addition, it is assumed that motivation increases the number of ideas produced and that the number of novel and useful ideas increases proportionately (for review, see Flaherty, 2005). Furthermore, motivation increases CPMDT (Halpin and Halpin, 1973). Although a wide range of mood and emotional states is thought to be important for CPMDT (Baas et al., 2008); among mood states, motivational state (state with full vigor and vitality) is shown to be particularly important for CPMDT. And there is a rather distinct positive association between CPMDT and higher motivational state (Takeuchi and Kawashima, 2013). On the other hand, emotional intelligence (EI) is defined as “*the subset of social intelligence that involves the ability to monitor one's own and others' feelings and emotions, to discriminate among them and to use this information to guide one's thinking and actions*” (Salovey and Mayer, 1990, p. 189). In addition, EI is known to promote better mood and emotional states (Uchiyama et al., 2001) including motivational state (Extremera and Fernández-Berrocal, 2005). In addition, the theoretical models of EI assume that motivation or the ability to motivate oneself is an essential part of EI (Goleman, 1998; Uchiyama et al., 2001). On the contrary, some theories suggest that higher EI leads to higher creativity (Mayer et al., 1999). Theoretically, it was also assumed that EI facilitates positive mood, which in turn facilitates creative thinking (Ivcevic et al., 2007). CPMDT has been shown to be positively associated with EI (Guastello et al., 2004). Given the aforementioned distinct association between CPMDT and motivational state, motivational state may be the link between CPMDT and EI.

Previous neuroscientific studies have shown that the dopaminergic system plays an important role in CPMDT, emotional control, and motivational state. A wide range of evidence has established the role of dopamine in motivation (Carlson, 2001). With regard to creativity or creative potential, recent neuroimaging studies have shown an association between CPMDT and dopamine receptor binding potential (De Manzano et al., 2010). Furthermore, mean diffusivity (MD) in the areas of the dopaminergic system, which is associated with dopamine synthesis capacity (Kawaguchi et al., 2014), displays an association with CPMDT (Takeuchi et al., 2015). These findings are congruent with the theory based on a wide range of evidence stating that the dopaminergic neural system may facilitate creativity through motivation as well as other dopamine-dependent cognitive processes, such as goal-directed thoughts and seeking behaviors (Flaherty, 2005). Finally, given the aforementioned essential link between motivation and EI, the dopamine neural system may also be theoretically linked to EI. Finally, the polymorphism of the dopamine D2 receptor (DRD2) gene is shown to be associated with emotional control (Blasi et al., 2009). Dopamine D2 function has been shown to be important for regulatory self-control (Pattij et al., 2007). While deficit EI has been shown to underlie disorders involving addiction or substance abuse, such as alcohol dependence (Schutte et al., 2011), so is the dopamine D2 function (Volkow et al., 2006).

One way to look at dopamine D2 function is to consider the polymorphism of the DRD2 gene. Among these, the Taq1A polymorphism (rs1800497) of the DRD2 gene is a substitution located in a noncoding region of the DRD2 locus. The A1 allele (as opposed to the A2 allele) of this polymorphism was shown to be robustly associated with alcohol dependence through a meta-analysis (Munafo et al., 2007). A previous meta-analysis of the association between this DRD2 polymorphism and substance dependence (Munafò et al., 2009). Furthermore, a physiological study (Thompson et al., 1997) revealed that the effect of this polymorphism has sex differences on dopamine physiology; therefore, the interaction effects between the DRD2 polymorphism and sex on phenotypes may exist. Moreover, for processing emotion and motivation as well as emotional and motivational responses to stimuli, sex differences are known to exist (e.g., Willner et al., 1998; Bradley et al., 2001). Additionally, there are sex differences in mood disorders (Kessler et al., 1993). Furthermore, concerning dopamine release and dopamine binding potential, sex differences are known to exist (Andersen and Teicher, 2000; Munro et al., 2006). Moreover, sex differences are also known to exist concerning the effects of polymorphisms that are related to emotions (Walderhaug et al., 2007).

From these lines of evidence, we hypothesized that dopamine D2 functional difference measured by DRD2 Taq1A polymorphism, EI, CPMDT and motivational states are associated with one another and each component mediates the others' associations. And we also assumed possible interaction effects between the DRD2 polymorphism and sex on other variables. As described previously, the associations among EI, CPMDT, and motivational state have been well established, and other polymorphisms of DRD2 known to be linked to emotion control have been previously established. However, the following remain unknown: (a) the associations of the DRD2 Taq1 polymorphism with EI, CPMDT, and motivational state, (b) these associations' possible sex differences, and (c) the mechanism by which the DRD2 Taq1 polymorphism, EI, CPMDT, and motivational state affect one another in the causal pathway. The purpose of this study was to reveal these mechanisms.

## Materials and methods

### Subjects

Data from 766 healthy, right-handed individuals (426 men and 340 women; 20.7 ± 1.9 years of age) were used in this study as a part of an ongoing project, consisting of various types of MRI scanning and psychological test batteries besides the ones analyzed in this manuscript, to investigate associations among brain imaging, cognitive functions, aging, genetics, and daily habits (Takeuchi et al., 2012a,2013b,c, 2014a,b). The description of the basic information of subjects in this study, was reproduced from our previous study (Takeuchi et al., 2013d, p. 320). All subjects were university, college, or post-graduate students or subjects who had graduated from these institutions within 1 year before the experiment and had normal vision. None had a history of neurological or psychiatric illness. Handedness was evaluated using the Edinburgh Handedness Inventory (Oldfield, 1971). This study was approved by the Ethics Committee of Tohoku University. Written informed consent was obtained from each subject and for nonadult subjects, written informed consent was obtained from the parent (guardian) of each subject by signing a form and in accordance with the World Medical Association (1991).

### Divergent thinking assessment

The methods outlined here are reproduced from our previous studies (Takeuchi et al., 2010a, pp. 12–13; 2010b, pp. 579–580; 2011a, p. 682; 2011b, p. 2; 2012b, pp. 2923–2924).

The S-A creativity test (Society_for_Creative_Minds, 1969) was used to assess CPMDT. J.P. Guilford generated the draft plan of this test. He also supervised the development of the test (Society_for_Creative_Minds, 1969). The test was standardized for Japanese speakers (Society_for_Creative_Minds, 1969).

The test is used to evaluate verbal CPMDT (Society_for_Creative_Minds, 1969), and it involves three types of tasks. The practice (and real) tasks are administered in the following order: (1) practice of the first task (2 min), (2) the first task (5 min), (3) practice of the second task (2 min), (4) the second task (5 min), (5) practice of the third task (2 min), and (6) the third task (5 min). Each task involves two questions. In total, the test takes 30 min. This test was administered in a group setting. The first task requires subjects to generate unique ways of using typical objects (e.g., “Other than reading, how can we use newspapers?” An example answer is “We can use them to wrap things.”). The second task requires subjects to imagine desirable functions of ordinary objects (e.g., “What are the characteristics of a good TV? Write down as many characteristics as possible.” An example answer is “A TV can receive broadcasts from all over the world.”). The third task requires subjects to imagine the consequences of “unimaginable things” happening (e.g., “What would happen if all the mice in the world disappeared?” An example answer is “The world would become more hygienic.”). Each task requires subjects to generate as many answers as possible. The S-A creativity test provides a total score, which was used in this study, as well as scores for the following dimensions of the creative process: (a) Fluency: Fluency is measured by the number of relevant responses to questions and is related to the ability to produce and consider several alternatives. Fluency scores are determined by the total number of questions answered after excluding inappropriate responses or responses that are difficult to understand. (b) Flexibility: Flexibility is the ability to produce responses from a wide perspective. Flexibility scores are determined by the sum of the (total) number of category types to which the responses are assigned based on a criteria table or an almost equivalent judgment. (c) Originality: Originality is the ability to produce ideas that differ from those of others. Originality scoring is based on the sum of idea categories that are weighted based on a criteria table or an almost equivalent judgment. (d) Elaboration: Elaboration is the ability to produce detailed ideas (Society_for_Creative_Minds, 1969). Elaboration scores are determined by the sum of responses that are weighted based on a criteria table or an almost equivalent judgment. These four dimensions correspond to the same concepts as those of the Torrance tests of creative thinking (TTCT; Torrance, 1966).

The total score is the sum of the originality score and that of elaboration in the version of the S-A creativity test (Society_for_Creative_Minds, 1969) used here. This is because the Fluency and Flexibility scores are highly correlated with those of Elaboration (Society_for_Creative_Minds, 1969). Scoring of the tests was performed by the Tokyo Shinri Corporation.

The analysis was limited to the total score, and it did not include the score for each dimension. This is because in this test, the score of each dimension is highly correlated with the total score and with those of other dimensions (Takeuchi et al., 2010a). This phenomenon is consistent with another similar divergent thinking test (Heausler and Thompson, 1988), namely TTCT (Torrance, 1966). Heausler and Thompson (1988) concluded that the correlations among the subscales in TTCT are so high that each subscale could not meaningfully provide dissociated information. Treffinger (1985) also warned that separate interpretations of TTCT subscores should be avoided. Consistent with this notion, a previous study (Chávez-Eakle et al., 2007) that investigated the association between regional cerebral flow (rCBF) and each dimension revealed that different dimensions were correlated with rCBF in similar regions. Thus, we believe that using only the total score serves the purpose of this study. However, another study using different approaches found two-factor structures in the subscales of figural TTCT, which contains six subscales (Kim, 2006). Furthermore, a previous study of the association of polymorphisms of dopamine-related genes and CPMDT found significant associaitons in some subscales but not in others, although whether the patterns of the results are statistically significantly different between different subscales is not clear from the report (Runco et al., 2011). In light of these findings, we assessed whether the correlations between the DRD2 polymorphism (for details, see the subsection below) and the four subscale scores were different in this study. The zero-order correlation coefficients of these correlations ranged from 0.086 to 0.10 in females and from −0.01 to 0.02 in males. Thus, apparently, there were no statistically distinguishable differences among the results of the correlation analyses between the four subscales of this test and the DRD2 polymorphism in this study.

Please refer to the Appendix for a sample and the manner in which the tests were scored.

For the information of the external validity of this scale, we quote our previous study (Takeuchi et al., 2010b, p. 579). S-A creativity test scores are significantly correlated with various other external measures, such as various personality factors and problem-solving abilities in daily life, suggesting its ability to predict performance in everyday situations (Shimonaka and Nakazato, 2007). Furthermore, S-A creativity test scores are significantly correlated with the frequency of visual hypnagogic experiences, which in turn are correlated with the vividness of mental imagery and neuroticism (Watanabe, 1998).

### Emotional intelligence scale

The methods outlined here are reproduced from our previous studies (Takeuchi et al., 2011c, p. 1499; 2013d, p. 320; 2013e, pp. 1026–1027).

The Japanese version of the EI scale (EIS) (Fukunishi et al., 2001b; Uchiyama et al., 2001) was used to assess EI as it was in our previous studies (Takeuchi et al., 2011c,2013d,e). The Emotional Intelligence Scale is a self-reported measurement that provides an estimate of emotional and social intelligence. The scale was developed and standardized for use with Japanese subjects. The Emotional Intelligence Scale comprises 65 items and a five-point Likert scale with a response format ranging from “not true of me” to “very often true of me.” The subjects' responses were categorized into the following three composite scale scores (factors): (a) intrapersonal factor (comprised of self-insight, self-motivation, and self-control), (b) interpersonal factor (comprised of empathy, altruism, and interpersonal control), and (c) situation management factor (comprised of insight into and control over a situation). Each composite scale score is composed of three subscale scores.

The intrapersonal factor evaluates (1) self-awareness, (2) the ability to sustain one's behavior, and (3) the ability to act appropriately. The interpersonal factor evaluates the ability to maintain appropriate personal relationships based on the understanding and empathy toward another person's emotions. The situation management factor evaluates (1) the ability of an individual to endure and adapt to a change, (2) provide leadership, and (3) exhibit flexibility in the control and use of their abilities in dynamic situations.

The following are examples of items on the Emotional Intelligence Scale.

“I know when my emotions change” (self-insight subscale in the Intrapersonal factor).

“I do not want to say something that offends someone else” (altruism subscale in the Interpersonal factor).

“I can respond to situational changes effectively” (control toward situation subscale in the Situation Management factor).

Other than this three-component model of EI, there are a four-component model of EI (Salovey and Mayer, 1990) and a five-component model of EI (Bar-On, 1997). The Bar-On model of EI (Bar-On, 1997) consists of two major factors, an intrapersonal and an interpersonal factor, as well as other minor factors, such as stress coping, adaptability, and general mood. On the contrary, based on a literature review, Otake et al. (2001) proposed a third major factor (situation management), which is equal to the minor factors of the Bar-On model. Basically, they proposed that EI is not limited to the self-related abilities and other-related abilities which previous models consistently included, and they proposed a factor to manage the situation. Moreover, based on these models, items were gathered and described. Then, based on the factor analyses, these three factor models were supported (Uchiyama et al., 2001).

The Emotional Intelligence Scale is an established test based on normative data with a large sample size (*n* = 703) (Uchiyama et al., 2001). The scoring of each factor is based on a test manual. Confirmatory factor analyses validate the model of this test (Otake et al., 2001; Uchiyama et al., 2001). According to the test manual (Uchiyama et al., 2001), the internal consistencies of the three factors (intrapersonal, interpersonal, and situation management factors) are 0.894, 0.915, 0.915 respectively (Cronbach's coefficient alpha).

In this study, we used the total score (sum of the three factors) of EIS, as in the case of the previous study (Takeuchi et al., 2013d). The previous study suggested that the polymorphism of EI is associated with not only aspects of self control (Blasi et al., 2009) but also social and situational aspects (Ponce et al., 2003). We therefore focused on the total EI score in this study. The associations of each factor with motivational state, CPMDT, and the DRD2 Taq1 polymorphism were highly similar and could not be statistically differentiated.

Scores on the Emotional Intelligence Scale are associated with EI related measurements such as the Toronto Alexithymia Scale (Fukunishi et al., 2001a). This indicates the external validity of the Emotional Intelligence Scale. All three factors of the Emotional Intelligence Scale are associated with improved mental health as determined by a general health questionnaire as well as increased optimism as determined by the LOT Optimism scale (Uchiyama et al., 2001). Specifically, the situation management factor was strongly associated with better mental health (Uchiyama et al., 2001). These results are consistent with the idea that higher TEI leads to better mental health (Salovey et al., 2000).

### Profile of mood states

Vigor subscale of the shortened Japanese version (Yokoyama, 2005) of the Profile of Mood States (POMS) (McNair et al., 1992), which measures participants' motivation, was used. In this study, we used the score of each participant's experience of mood during the week preceding the experiment (Takeuchi et al., 2011b) (which means the experience of the mood on the day of the experiment as well as that during the past week before the experiment). Cronbach's alpha of this subscale is 0.869 (Yokoyama, 2005). The score of this subscale is decreased in a number of diseases and after exhausting work (Yokoyama, 2005).

### Genotyping of DRD2/ANKK1 Taq1A polymorphism

High-molecular-weight DNA was isolated from the saliva of subjects using Oragene containers (DNA Genotek Inc., Canada), according to the manufacturer's protocol. DRD2/ANKK1 Taq1A polymorphism (rs1800497) was genotyped utilizing the Taqman Allelic Discrimination Assay System (assay ID: C_7486676_10) obtained from Applied Biosystems (Foster City, CA, USA). Each genomic DNA (20 ng) was mixed with 0.25 ml of primer/TaqMan Probe mixture and 5 ml of TaqMan Universal PCR Master Mix (Applied Biosystems) within 10 ml of the total volume. Thermal cycling conditions were 95°C for 10 min, followed by 50 cycles of 92°C for 15 s and 59°C for 1 min in the CFX96 Real-Time System (BioRad, Hercules, CA, USA). Alleles were determined on the basis of allelic discrimination features of the CFX Manager software (BioRad). qRT-PCR-based genotyping data was validated on the basis of sequencing of PCR products (635 bp) of representative subjects, utilizing the following primers: forward: ccctgcatctagcagcctac, reverse: gagacagggttttgccatgt, spanning the polymorphic site.

DRD2 was coded A1/A1, A1/A2, and A2/A2. Among the 778 participants whose psychological and genetic data were obtained in this study, data for the polymorphism were successfully obtained from 766 subjects (426 men and 340 women; 20.7 ± 1.9 years of age); genotyping data of 12 subjects were not available because of failures either in proper extraction of a DNA sample from the saliva or in amplification in the PCR procedure or failure to provide a (proper) saliva sample. The genotypic distributions of the 766 subjects were as follows: DRD2 Taq1A A1/A1 (men, *n* = 57, 7.4%; women, *n* = 37, 4.8%), DRD2 Taq1A A1/A2 (men, *n* = 197, 25.7%; women, *n* = 153, 20.0%), and DRD2 Taq1A A2/A2 (men, *n* = 172, 22.5%; women, *n* = 150, 19.6%). Allele frequencies of A1 and A2 alleles were 35.1% and 64.9%, respectively, which were concordant with previous findings (Tsuchimine et al., 2012). Tests for the Hardy–Weinberg equilibrium exhibited no deviations from the expected genotype distribution (*p* > 0.05).

As described in the previous study (Stice et al., 2010), the DRD2 Taq1A site exists in exon 8 of the ANKK1 gene on the opposite strand. This SNP results in a glutamate-to-lysine (E713K) substitution within the eleventh ankyrin repeat of ANKK1. This suggests that changes in the function of ANKK1 may be relevant to some associations that are attributed to DRD2 (Neville et al., 2004). Keeping this in mind, we refer to the polymorphism as DRD2 Taq 1A in this study.

### Statistical analyses of the effects of the DRD2 Taq1A polymorphism

Behavioral data were analyzed using SPSS 22.0 (SPSS Inc., Chicago, IL). First, the associations between the DRD2 Taq1A polymorphism (DRD2 Taq1A A1/A1 = 1, DRD2 Taq1A A1/A2 =2; DRD2 Taq1A A2/A2 = 3) and the scores for the cognitive measures that were common to both sexes were analyzed using multiple regression analyses. Additional covariates for each analysis were age and sex. Second, the interaction effects between sex and the DRD2 Taq1A polymorphism on cognitive measures were analyzed using analyses of covariance (ANCOVAs). Sex was a fixed factor, and additional covariates were the DRD2 Taq1A polymorphism and age. These three variances and the interaction between sex and the DRD2 Taq1A polymorphism were included in the model. Finally, associations between the DRD2 Taq1A polymorphism (DRD2 Taq1A A1/A1 = 1, DRD2 Taq1A A1/A2 = 2; DRD2 Taq1A A2/A2 = 3) and the scores for the cognitive measures in each sex were analyzed using multiple regression analyses with age as a covariate.

In psychological analyses, results with a threshold of *p* < 0.05, corrected for false discovery rate (FDR) using the two-stage sharpened method (Benjamini et al., 2006), were considered statistically significant. The correction for multiple comparisons using this method were applied to the results of abovementioned three ANCOVAs (analyses for interactions between sex and DRD2 Taq1A polymorphism on Vigor subscale of POMS, the total score of EIS, and the score of S-A creativity test) and 18 multiple regression analyses (analyses for associations between two of the polymorphism of DRD, Vigor subscale of POMS, the total score of EIS, and the score of S-A creativity test for both sexes, men, women).

### Path analysis of the associations between the DRD2 Taq1A polymorphism, EI, CPMDT, and motivational state

The results of analyses described above suggested that there were sex differences in the associations between the polymorphism of DRD2 and psychological variables. There were also associations among the DRD2 Taq1A polymorphism on the Vigor subscale of POMS, the total score of EIS, and the score of the S-A creativity test in females.

We then proceeded to path analyses for identifying the association among these variables, particularly in females. As described in a previous study (Charlton et al., 2008), structural equation modeling (SEM) was used to simultaneously estimate the relationships among the abovementioned four variables. Intercepts were allowed in the structural equations, and models were fitted using maximum likelihood methods. SEM was performed using the Amos software (version 22.0, IBM, SPSS). We included the abovementioned four variables.

**Figure 1:**
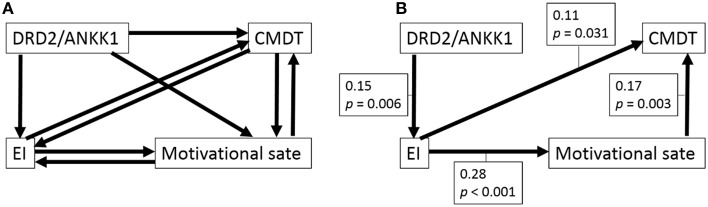
**Path analysis of the associations among the polymorphism, CPMDT, motivational state, and emotional intelligence in females. (A)** The initial model is shown. Depending on the directionality of the paths among four variables, there were eight initial models. From each initial model, the paths with the highest *P*-value were deleted recursively one by one, the analyses were rerun after each path was removed, until the model fit stopped improving. **(B)** The final model is shown. Standardized regression weights for the significant paths and *P*-values are shown next to each path arrow.

**Figure 2:**
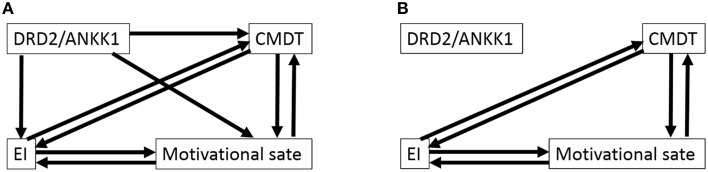
**Path analysis of the association among the polymorphism, CPMDT, motivational state, and emotional intelligence in males. (A)** The initial model is shown. Depending on the directionality of the paths among four variables, there were eight initial models. From each initial model, the paths with the highest *P*-value were deleted recursively one by one, and analyses were rerun after each path was removed, until the model fit stopped improving. **(B)** The final model is shown. Depending on the directionality of the paths among personalities, there were eight final models with equal statistical values.

In constructing the initial models, we assumed that the polymorphism affected the psychological variables and not the other way around. We did not make any further assumptions in this study. Thus, there were eight initial models for each sex because we could not presume the direction of paths between psychological variables (Figures 1A, 2A). Subsequently, as described in the previous study (Charlton et al., 2008), we considered whether the paths of relatively complex literature-derived models shown in Figures 1A, 2A could be reduced by removing pathways that lacked statistically significant associations and the models could be improved. To obtain a better model, we employed stepwise removal or alternation procedure that fitted the model, as described in the previous studies (Charlton et al., 2008; Fjell et al., 2012). The models were evaluated by comparing the fit of nested models that included and excluded a path in question and by using Akaike information criterion (AIC) and statistics of fitness. Once a final model was obtained, regression coefficients were estimated for all the remaining paths. To check that the final model fitted the data adequately, two verifications were performed: (a) a test to check for the lack of fit was performed using chi-squared statistics, and (b) the following fit indices were calculated: AIC, the comparative fit index (CFI), and the root mean square error of approximation (RMSEA).

Based on the initial models, the paths with the highest *P*-value were deleted recursively one-by-one, and the analyses were rerun after each path was removed until the model fit stopped improving.

## Results

### The basic demographic and psychological characteristics of each genotype

The basic demographic variables and psychological variables of each genotype and each sex are presented in Table 1.

**Table 1:** **The descriptive data for each genotype of the DRD2 Taq1 polymorphism in each sex**.

	**Men**			**Women**		
	**DRD2-Taq1**	**DRD2-Taq1**	**DRD2-Taq1**	**DRD2-Taq1**	**DRD2-Taq1**	**DRD2-Taq1**
	**A1/A1**	**A1/A2**	**A2/A2**	**A1/A1**	**A1/A2**	**A2/A2**
	**(*n* = 57)**	**(*n* = 197)**	**(*n* = 172)**	**(*n* = 37)**	**(*n* = 153)**	**(*n* = 150)**
Age	20.68±2.14	20.71±1.86	20.91±2.01	20.16±1.48	20.51±1.61	20.68±1.71
S-A creativity test	36.88±10.94	35.96±9.61	36.19±10.97	35.51±7.54	38.00±9.72	39.09±10.02
Vigor subscale of POMS	8.37±3.8	8.51±3.86	8.12±3.97	7.14±3.59	7.86±3.83	8.40±4.18
Total score of EIS	133.39±31.17	127.22±33.82	126.61±37.38	123.03±30.13	124.56±36.84	134.83±28.77

### The associations among psychological variables and the polymorphism of DRD2 across sexes

The associations among the EIS score, Vigor subscale score of POMS, score of the S-A creativity test, and DRD2 Taq1 polymorphism were tested using multiple regression analyses correcting for age and sex. The statistical values are presented in Table 2. The correlations between (a) the EIS score and S-A creativity test score, (b) the EIS score and the score of the Vigor subscale of POMS, and (c) the S-A creativity test score and Vigor subscale of POMS were significant, and were all positive correlations. However, the correlations of the DRD2 polymorphism with other psychological scores were not significant. Note that this result (c) is reported using the smaller sample in this project (Takeuchi et al., 2013a).

**Table 2:** **Statistical values (beta-value, *t*-value, uncorrected *P*-value, *P*-value corrected using FDR) of the multiple regression analyses in men, women (controlling for age), and the entire sample (controlling for age and sex)**.

**Dependent variables**	**Independent variables**	**Male**				**Female**				**All**			
		**β**	**t**	**P (unc)**	**P (FDR)**	**β**	**t**	**P (unc)**	**P (FDR)**	**β**	**t**	**P (unc)**	**P (FDR)**
S-ACT^a^	Vigor^b^	0.198	4.149	4.030 × 10^−5^	6.045 × 10^−5^	0.173	3.203	0.001	0.001	0.187	5.232	2.169 × 10^−7^	4.554 × 10^−7^
S-ACT	EIS^c^	0.301	−6.573	1.454 × 10^−10^	−5.089 × 10^−10^	0.133	2.446	0.015	0.014	0.231	6.571	9.283 × 10^−11^	4.874 × 10^−10^
S-ACT	DRD2^d^	−0.013	−0.277	0.782	0.391	0.097	1.777	0.076	0.057	0.034	0.932	0.351	0.217
Vigor	EIS	0.295	6.278	8.518 × 10^−10^	2.236 × 10^−9^	0.261	4.927	1.311 × 10^−6^	2.294 × 10^−6^	0.280	7.972	5.713 × 10^−15^	5.999 × 10^−14^
Vigor	DRD2	−0.034	−0.696	0.487	0.269	0.097	1.785	0.075	0.057	0.024	0.654	0.513	0.269
EIS	DRD2	−0.059	−1.229	0.220	0.144	0.140	2.587	0.010	0.011	0.025	0.672	0.502	0.269

a *S-A creativity test score*.

b *Vigor subscale score of POMS*.

c *Total score of EIS*.

d *DRD2 Taq1 polymorphism (number of A2 allele)*.

### The effects of the interaction between sex and the polymorphism on psychological variables

ANCOVA with age as a covariate revealed the significant effect of the interaction between sex and the polymorphism of the DRD2 gene on the total score of EIS (*F* = 7.114, uncorrected *P* = 0.008, *P*-value corrected for FDR in a studywise manner = 0.009) but not on the score of the Vigor subscale of POMS (*F* = 3.269, uncorrected *P* = 0.071, *P*-value corrected for FDR in a studywise manner = 0.057) and the score of the S-A creativity test (*F* = 2.460, uncorrected *P* = 0.117, *P*-value corrected for FDR in a studywise manner = 0.082). For the descriptive data, see Table 1.

### The association among psychological variables and the polymorphism of DRD2 in each sex

*Post-hoc* multiple regression analyses using data from either one of two sexes revealed that there was a significant relationship between the polymorphism and the total score of EIS only in females in that the number of A2 alleles was significantly and positively correlated with the total score of EIS. In addition, for both sexes, significant associations were found between any two of the total score of EIS, the score of the Vigor subscale of POMS, and the score of the S-A creativity test. For statistical values, see Table 2.

### Path analysis involving CPMDT, motivational state, EI, and the DRD2 polymorphism

The eight initial models created to generate the model involving CPMDT, motivational state, EI, and the DRD2 polymorphism were presented in Figure 1A (males) and Figure 2A (females).

For females, from each initial model (AIC = 28, CFI = 1.0, RMSEA = 0.111), the paths with the highest *P*-values were deleted recursively one by one, and analyses were rerun after each path was removed, until the model fit stopped improving. One of the final models showed the best statistic for the fit of the models (Figure 1B, chi-squared statistic = 3.284, *df* = 2, *P* = 0.194, AIC = 27.284, CFI = 0.973, RMSEA = 0.041). In this final model, all the paths reached significance. In this final model, (a) the DRD2 polymorphism primarily facilitated EI (the total score of EIS), (b) EI in turn facilitated CPMDT (the score of the S-A creativity test) and led to a better motivational state (the score of the Vigor subscale of POMS), and (c) the better motivational state also directly facilitated CPMDT.

For males, from each initial model (AIC = 28, CFI = 1.0, RMSEA = 0.142), the paths with the highest *P*-values were deleted recursively one by one, and analyses were rerun after each path was removed, until the model fit stopped improving. The three paths from the DRD2 polymorphism were removed. However, the directions of the paths among the three psychological variables could not be determined and there were eight final models that showed the same statistics for the fit of the models (Figure 2B, chi-squared statistic = 1.303, *df* = 3, *P* = 0.728, AIC = 23.303, CFI = 1.0, RMSEA < 0.001).

## Discussion

In this study, we demonstrated that there were significant positive relationships among CPMDT, EI, and motivational state across sex. In addition, the polymorphism of the DRD2 gene was significantly associated with EI, specifically in females. Path analysis in females indicated that the model in which (a) the DRD2 polymorphism primarily facilitates EI, (b) EI in turn facilitates CPMDT and leads to a better motivational state, and (c) the better motivational state also directly facilitates CPMDT explained the data in the most accurate manner. Thus, our hypothesis was at least partially confirmed in females. However, it should be noted that the results of path analysis do not prove that the finalized model is statistically significantly better than the other models and that it does not account for the variables that are not in the models. For males, the associations among EI, CPMDT, and a better motivational state were confirmed and the polymorphisms of DRD2 did not show an association with any of these factors.

This study depicted a comprehensive picture of the association among dopamine, EI, motivational state, and CPMDT at least in females. As described in the Introduction, the associations between two of these factors have been previously reported. These include the association between emotional regulation and the polymorphism of the DRD2 gene (Blasi et al., 2009), the association between EI and CPMDT (Guastello et al., 2004), the association between EI and better mood (Uchiyama et al., 2001), and the association between motivational state and CPMDT (Takeuchi et al., 2013a). In addition, we previously analyzed the iron mineral which is critical to dopamine processing and suggested that dopaminergic physiology is indirectly associated with CPMDT (Takeuchi et al., 2013a). We also showed that microstructural properties of the areas of the dopaminergic system are indirectly associated with CPMDT through personalities that are related to motivation (Takeuchi et al., 2015). The results of the present study are congruent with those of these previous studies and have advanced our understanding of the interactions among dopamine, EI, motivational state, and CPMDT. In particular, it has been theoretically assumed that motivation increases divergent thinking and creativity and not vice versa (for review, see Flaherty, 2005), and at least some theories suggest that EI facilitates positive mood, which in turn facilitates creative thinking (and not vice versa) (Ivcevic et al., 2007). In addition, it has been theoretically assumed that dopamine systems underlie these cognitive processes. The results in females are congruent with the theoretical views and empirically support these views. In addition, the twin study already demonstrated the role of the substantial contribution of genetics to EI (Vernon et al., 2008). In addition, our results extended this previous finding from the twin study, indicating that polymorphisms of dopamine-related genes may at least partly contribute to the genetics of EI.

One interesting speculation arising from this study is that in females, because the physiology of dopamine receptor D2 is associated with EI, by modulating this physiology through agonist and antagonist, we may be able to modulate EI. On the other hand, it has been shown that EI can be trained and consequently improve well-being (Slaski and Cartwright, 2003). Thus, through such training, we may be able to enhance CPMDT as well.

The studies of polymorphism suffer from low effect sizes of the polymorphism (Murphy et al., 2012), and this study may suffer from the same limitation. Although we focused on the polymorphism that is shown to have robust effects on the phenotypes through meta-analyses (Munafo et al., 2007; Munafò et al., 2009), the effect in females was not very marked and the effect in males was negative. To show the association between dopamine physiology and EI more robustly and in a brain region-specific manner, neuroimaging techniques such as dopamine receptor binding potential measures of positron emission tomography (PET) can be utilized (Hirvonen et al., 2004).

The possible mechanism of how the A1 allele of the DRD2 Taq1 polymorphism is associated with behaviors that are apparently related to less EI is summarized in a previous study (Bowirrat and Oscar-Berman, 2005). Basically, in this model, the A1 allele of this polymorphism is associated with less density of the DRD2 receptor. Substances of abuse such as alcohol and tobacco and other most positive reinforcers cause dopamine release in the brain, which can decrease negative feelings and satisfy cravings. The deficiency of the DRD2 receptor in people with the A1 allele reduces their capacity for enjoying this reward naturally and their requirement of positive reinforcers is greater and less controllable. These conditions lead to their addictive, impulsive, and compulsive behaviors. These descriptions correspond well with at least conditions of parts of less EI (Uchiyama et al., 2001) and explain well the negative impact of the A1 allele of the DRD2 Taq1 polymorphism on EI.

One possible speculation about why there was a female-specific effect of the DRD2 Taq1 polymorphism in the present sample is related to environmental and cultural backgrounds. As hypothesized in the Introduction, a previous meta-analytic study showed sex differences in the effects of the DRD2 Taq1 polymorphism on substance dependence (Munafò et al., 2009). However, in this case, the A1 allele was associated with the phenotype more strongly in males. On the other hand, other studies showed that the A1 allele was associated with phenotypes more strongly in females. For example, Lee et al. (2003) showed that in an Asian sample of their study (Korea), the A1 allele was associated with a higher reward dependency only in females. Related to this, one recent study using a huge sample showed that the effects of the DRD2 Taq1 polymorphism on the harshness of mothers' parenting are modulated by the economic situation; when the macroeconomic conditions are deteriorating, the A1 allele is associated with mothers' harsh parenting, but when it is not, the allele does not necessarily have such impact (Lee et al., 2013). These results suggest that the effects of the DRD2 polymorphism appear only under certain conditions such as stress. On the other hand, it has been said that in Japan, women are oppressed in many aspects and women from universities tend to feel more stressed than males (Suzuki et al., 1997). These cultural backgrounds may explain the present female-specific effects of the DRD2 Taq1 polymorphism. However, certainly, these are speculative possibilities that we propose to explain the present results. Future study is required to investigate this issue. In addition, it is generally known that sex differences in cognition are small when they exist (Zell et al., 2015). Furthermore, the measures we used hold true to such patterns (see Table 1). However, apparently, men and women are culturally exposed to different environments during development. Moreover, the environment and genes are assumed to interact and affect phenotype (Caspi and Moffitt, 2006). The aforementioned view may hold true to this pattern.

It should be noted that perhaps, the present finding of the positive associations among EI, CPMDT, and better motivational state does not apply to the group in the field of art. While a previous study has identified the positive relationship between EI and verbal divergent thinking using a large sample (Guastello et al., 2004), another previous study using the figurative divergent thinking test and a small sample revealed a negative relationship between divergent thinking performance and measures of EI in the field of art (but there was an opposite insignificant pattern in the groups of science) (Sánchez-Ruiz et al., 2011). In addition, a meta-analysis (Feist, 1998) revealed that personalities that lead to higher creativity in science and those of artists are at least partially different. For example, with regard to personalities related to EI, artists are characterized as personalities displaying less tolerance and less sociability, whereas creative scientists are the opposite. Our sample did not include the students belonging to the faculty of typical art. Thus, the model of the associations among the polymorphism, EI, motivational state, and CPMDT can be different in the group of arts.

This study had at least a few limitations. One was common to our previous studies and other studies that used college cohorts (Song et al., 2008; Jung et al., 2010; Takeuchi et al., 2010a,b) and since this problem is common across relevant studies. We quote our study (Takeuchi et al., 2011a, p. 686) for this matter. “*Limited sampling of the full range of intellectual abilities is a common hazard when sampling from college cohorts. However, given the correlation between intelligence and creativity among subjects with normal and inferior intelligence (but not subjects with higher intelligence,”* Another limitation is common to our previous studies using the S-A creativity test. To measure creativity, we only used the S-A creativity test, which is a measure of verbal CPMDT, and we did not use tests for figural divergent thinking. However, as summarized by Jung et al. (2010), and as we quoted previously (Takeuchi et al., 2015, p. 1823), “*several cognitive processes are important for creativity or creative measures, such as flow (Csikszentmihalyi, 1997), insight (Jung-Beeman et al., 2004), perseverance in the face of social acceptance or resistance, such as that of personality variables, creative achievements, and remote association of ideas.”* Divergent thinking tests are by far the most used measure in the field to measure creative potential (Dietrich, 2007), and their validity to predict creative achievement has been established (Kim, 2008). However, different creative processes and measures may exhibit different patterns of associations with variables used in the study. Future studies need to investigate this issue.

### Conflict of interest statement

The authors declare that the research was conducted in the absence of any commercial or financial relationships that could be construed as a potential conflict of interest.

## Appendix

We quote the manner by which the S-A creativity test is scored from our previous study (Takeuchi et al., 2010b, pp. 583–584) as follows:

“*This appendix presents sample answers to a problem in the S-A creativity test, and the manner in which they were scored.”*

Sample question: Other than for storing milk, how can we use milk bottles?

Sample answers:

1. *Make a hole in it and use it as a coin bank*.
2. *Use it as a weight*.
3. *Use it as an instrument*.
4. *Use it as an object for shooting*.
5. *Beat on it and make a sound*.
6. *Eat it*.

*The manner in which they were scored is as follows*.

1. *Inappropriate answers were excluded. In this case, the sixth answer (Eat it) was excluded*.
2. *If the answer is included in categories on the criteria table, the answer is categorized and elaborate scoring is performed based on the table. Each category has an originality score. In the criteria table, the score is determined on the basis of how rare the category of certain answers is (If the category of a certain answer appears in more than 5% of the answers, the category has 0 originality points. If the category of a certain answer appears in less than 5%, but more than 1% of the answers, the category has 1 originality point. If the category of a certain answer appears in less than 1% of the answers, the category has 2 originality points.)*.
3. *If the answer is not included in the categories on the criteria table, and if it cannot be considered to belong to the same category as any of the other answers that are not included in the categories on the criteria table, then it is categorized as a new category and scoring of Elaboration score is performed in a similar manner to that of the criteria table*.

*Scores of each answer are as follows*.

1. Make a hole in it and use it as a coin bank.”
  Category: To use it as a vessels
  Originality of the category = 0, Category number = 1, Elaboration = 2
2. *Use it as a weight*.
  Category: To use it as a measure or to use its shape or weight
  Originality of the category = 1, Category number = 2, Elaboration = 1
3. *Use it as an instrument*.
  Category: To use it as an instruments
  Originality of the category = 1, Category number = 3, Elaboration = 0
4. *Use it as an object of shooting*.
  Category: Others 1
  Originality of the category = 2, Category number = 4, Elaboration = 2
5. *Beat on it and make a sound*.
  Category: To use it as an instruments
  Originality of the category = 1, Category number = 3, Elaboration = 1
6. *Eat it*.
  Inappropriate answer
  *The total score of the sample answers are as follows*.
  *Fluency = Number of appropriate answers = 5*.
  *Flexibility = Number of different categories = 4*.
  *Originality = The sum of the originality scores of the different categories = 4. (In this case, category number 1 = 0 points, category number 2 = 1 point, category number 3 = 1 point, category number 4 = 2 points)*.
  *Elaboration = The sum of the elaboration scores of all the answers = 6*.
  *Total score = Originality + Elaboration = 10*.”
